# The Correlation Between SPP1 and Immune Escape of EGFR Mutant Lung Adenocarcinoma Was Explored by Bioinformatics Analysis

**DOI:** 10.3389/fonc.2021.592854

**Published:** 2021-06-10

**Authors:** Yi Zheng, Shiying Hao, Cheng Xiang, Yaguang Han, Yanhong Shang, Qiang Zhen, Yiyi Zhao, Miao Zhang, Yan Zhang

**Affiliations:** ^1^ Department of Oncology, Shijiazhuang People’s Hospital, Shijiazhuang, China; ^2^ Department of Cardiothoracic Surgery, Standford University, Stanford, CA, United States; ^3^ Department of Oncology, Affiliated Hospital of Hebei University, Baoding, China

**Keywords:** immune checkpoint inhibitors, lung adenocarcinoma, epidermal growth factor receptor, tumor microenvironment, secreted phosphoprotein 1, tumor-infiltrating immune cells

## Abstract

**Background:**

Immune checkpoint inhibitors have achieved breakthrough efficacy in treating lung adenocarcinoma (LUAD) with wild-type epidermal growth factor receptor (EGFR), leading to the revision of the treatment guidelines. However, most patients with EGFR mutation are resistant to immunotherapy. It is particularly important to study the differences in tumor microenvironment (TME) between patients with and without EGFR mutation. However, relevant research has not been reported. Our previous study showed that secreted phosphoprotein 1 (SPP1) promotes macrophage M2 polarization and PD-L1 expression in LUAD, which may influence response to immunotherapy. Here, we assessed the role of SPP1 in different populations and its effects on the TME.

**Methods:**

We compared the expression of SPP1 in LUAD tumor and normal tissues, and in samples with wild-type and mutant EGFR. We also evaluated the influence of SPP1 on survival. The LUAD data sets were downloaded from TCGA and CPTAC databases. Clinicopathologic characteristics associated with overall survival in TCGA were assessed using Cox regression analysis. GSEA revealed that several fundamental signaling pathways were enriched in the high SPP1 expression group. We applied CIBERSORT and xCell to calculate the proportion and abundance of tumor-infiltrating immune cells (TICs) in LUAD, and compared the differences in patients with high or low SPP1 expression and wild-type or mutant EGFR. In addition, we explored the correlation between SPP1 and CD276 for different groups.

**Results:**

SPP1 expression was higher in LUAD tumor tissues and in people with EGFR mutation. High SPP1 expression was associated with poor prognosis. Univariate and multivariate cox analysis revealed that up-regulated SPP1 expression was independent indicator of poor prognosis. GSEA showed that the SPP1 high expression group was mainly enriched in immunosuppressed pathways. In the SPP1 high expression group, the infiltration of CD8+ T cells was lower and M2-type macrophages was higher. These results were also observed in patients with EGFR mutation. Furthermore, we found that the SPP1 expression was positively correlated with CD276, especially in patients with EGFR mutation.

**Conclusion:**

SPP1 levels might be a useful marker of immunosuppression in patients with EGFR mutation, and could offer insight for therapeutics.

## Introduction

Lung cancer has become one of the most serious threats to human health, and its global morbidity and mortality rank first among all cancer types (1). Approximately 85% of lung cancers are non-small cell lung cancer (NSCLC), and lung adenocarcinoma (LUAD) accounts for 40%–50% of NSCLC. In China, 50%–60% of patients with LUAD also have epidermal growth factor receptor (EGFR) mutation. Epidermal growth factor receptor-tyrosine kinase inhibitor (EGFR-TKI) targeted therapy has been recommended for treating patients with EGFR sensitive mutations and such therapy has significantly improved survival in advanced NSCLC (2, 3). However, EGFR-TKI resistance has been observed in patients with NSCLC, which is challenging the prognosis of the disease (4).

Recently, immune checkpoint inhibitors (ICIs), represented by programmed cell death-1 (PD-1) and programmed cell death-ligand 1(PD-L1) monoclonal antibodies, have presented a new approach for NSCLC treatment (5, 6). ICIs achieve long-term disease control in patients who have developed an anti-tumor response, by activating the body’s immune system for tumor cell recognition and removal (7). However, ICIs had poor efficacy and adverse effects in patients with EGFR mutation or secondary T790M mutation (8–10).

The IMpower 150 study found that a combination of atezolizumab, bevacizumab, and chemotherapy improved overall survival (OS) in patients with EGFR mutation (11). This study was the first randomized phase III trial of immunotherapy that showed a benefit in patients with EGFR mutation, suggesting that “primary drug resistance” could be reversed. *In-vitro* studies showed that the non-inflammatory tumor microenvironment (TME) changed in EGFR-mutated NSCLC after partial drug intervention, improving the efficacy of immunotherapy (12). Hence, there might be a connection between the EGFR-mutated NSCLC immune microenvironment and the mechanism of primary resistance to ICIs. Exploring the microenvironment characteristics may improve our knowledge of drug resistance mechanism and offer clues to reversing resistance.

Osteopontin (OPN, encoded by SPP1) is a secreted phosphorylated glycoprotein, which is produced by T, NK and other immune cells, myeloid cells, osteoblasts, bone cells, epithelial cells, etc. (13, 14). It is also a kind of multifunctional cytokine. Previously, we found that lung adenocarcinoma cells induced M2 polarization of macrophages through SPP1, and activation of T cells were observed after SPP1 silencing (15). However, the role of SPP1 mediated immunosuppression in patients with EGFR mutations remains unclear. What is more, the B7 family is important in regulating T cell immune response. PD-1 and B7-H3 (CD276) are both members of B7/CD28 family and had similar effects in TME (16). The up-regulated of CD276 expression can promote immune escaping of tumor cells, including inhibit the proliferation of T cells, reduce the secretion of IFN-γ, tumor necrosis factor-alpha (TNF-α), and other cytokines (16). As a co-inhibitory molecule of T cell, CD276 is an attractive target for cancer immunotherapy (17, 18).

**Figure 1:**
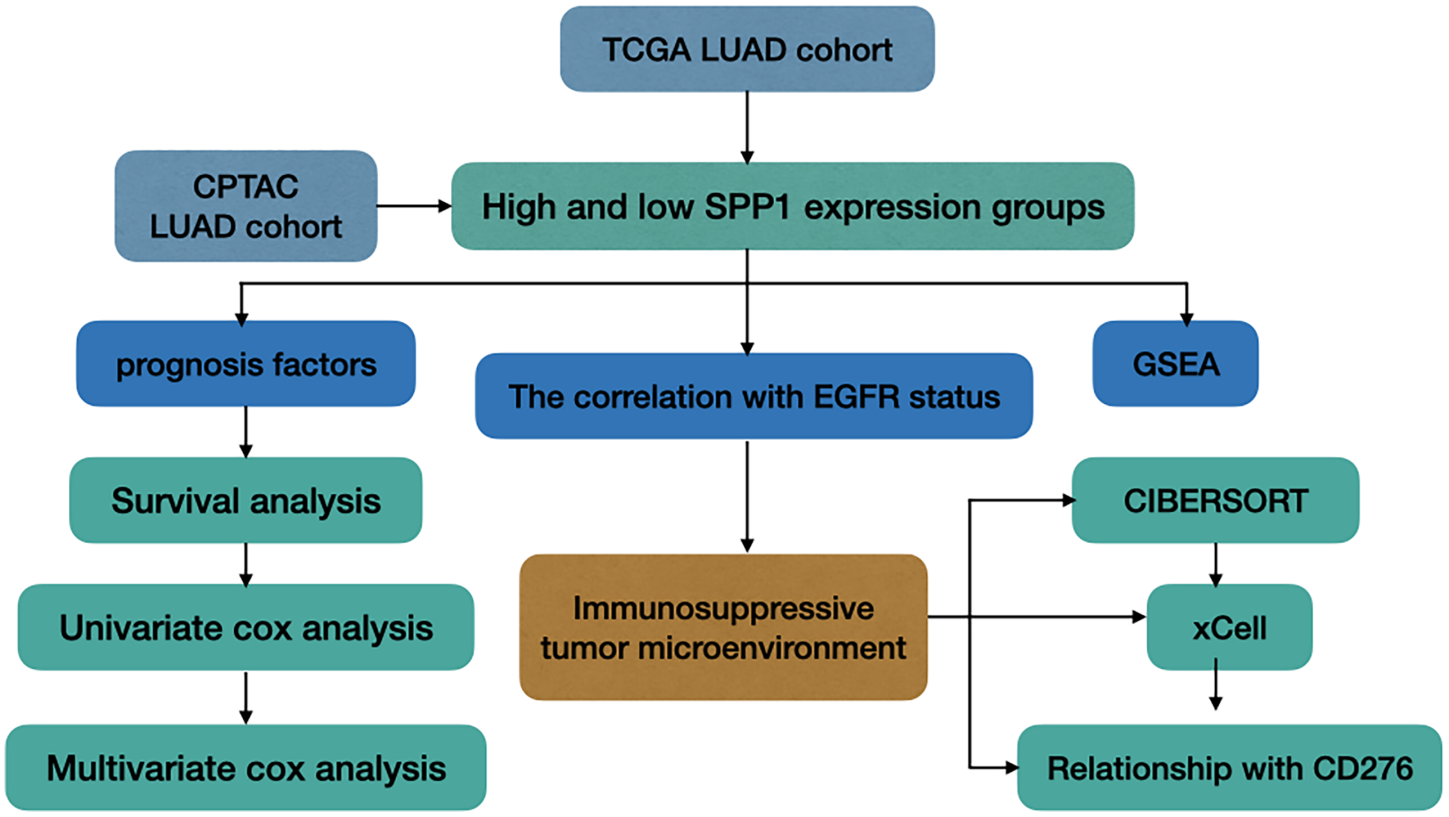
Study workflow.

In this study, we explored the TME of patients with EGFR mutation, which has been highlighted for its potential impact on the resistance of ICIs. We analyzed SPP1 expression in LUAD with or without EGFR mutation, and explored its association with clinicopathologic characteristics and patient outcomes. We further evaluated the differences in the tumor-infiltrating immune cells (TICs) in the immune microenvironment between groups with different levels of SPP1 expression, and those with or without EGFR mutation. In addition, the correlation between SPP1 and CD276 for different groups was compared. A study workflow is presented in **Figure 1**.

## Materials and Methods

### Data Acquisition

LUAD patient datasets were downloaded from The Cancer Genome Atlas (TCGA, https://portal.gdc.cancer.gov/) (19, 20), including transcriptome RNA-seq gene expression profiles (Level 3), and clinical information. A total of 497 tumor tissues and 54 adjacent tissues were included. We compared SPP1 protein expression in 102 normal and 109 cancer tissues, using The Clinical Proteomic Tumor Analysis Consortium (CPTAC, https://proteomics.cancer.gov/programs/cptac) (21, 22). We collected the mutation information of TCGA cohort from UCSC Xena database (https://xenabrowser.net/datapages/) (23), in which there are 472 cases with mutation information available including 409 EGFR wild-type and 63 EGFR mutant cases. Additionally, survival of patients with LUAD was analyzed in relation to SPP1 expression in TCGA and CPTAC databases.

### Cox Regression Analysis and GSEA

Univariate and multivariate cox regression analysis was performed in 435 patients (missing clinical information were excluded) to screen factors significantly associated with OS in TCGA. Gene Set Enrichment Analysis (GSEA) (http://software.broadinstitute.org/gsea/) was performed to determine the biological differences and pathways affected by differential expression of SPP1 (one-fourth cutoff) in Gene Set c2(c2.cp. Kegg.v7.0.symbols) and c5(c5.all.v7.0.symbols). The number of random sample permutations was set at 1000. The significance threshold was *P *< 0.05 and false discovery rate (FDR) < 0.25.

### Correlation With TICs and CD276

CIBERSORT(http://cibersort.stanford.edu/) is an immune cell infiltrating assessment analysis tool (24, 25). We assessed the influence of SPP1 expression and EGFR mutation to 22 types of immune cells by CIBERSORT. The filter criteria of each sample is set as the *P* < 0.05, which indicating that the inferred proportion of each TICs subtype are accurate and suitable for further analysis. We calculated the correlation between different immune cells, and marked those with *P* < 0.05. xCell is a gene signatures-based method, which performs cell type enrichment analysis from gene expression data for 64 immune and stroma cell types (26). To verify the results of CIBERSORT, we downloaded the results of immune cell abundance in TCGA LUAD by xCell algorithm from TIMER database (http://timer.cistrome.org/) (27). Besides, the correlation between SPP1 and CD276 for different groups was calculated using Spearman correlation coefficients.

### Statistical Analysis

Statistical analysis of data from TCGA and CPTAC were performed using R-3.6.1. The independent samples t-test or Wilcoxon’s rank sum-tests were used to compare continuous variables between two groups. Kruskal-Wallis one-way analysis of variance followed by a posthoc Kruskal-Dunn test with BH’s method for adjusting for multiple comparisons. Prism8 software was used to plot the survival curves using the Kaplan-Meier method, and the log-rank test was used to compare the survival curves. Uni- and multi-variate analyses were performed using Cox proportional hazard models, where *P* < 0.05 was considered statistically significant. The correlation between SPP1 and CD276 was calculated using Spearman’s correlation coefficient (R), with *P* < 0.01 was considered statistically significant.

## Results

### SPP1 Expression and OS Differences

**Figure 2:**
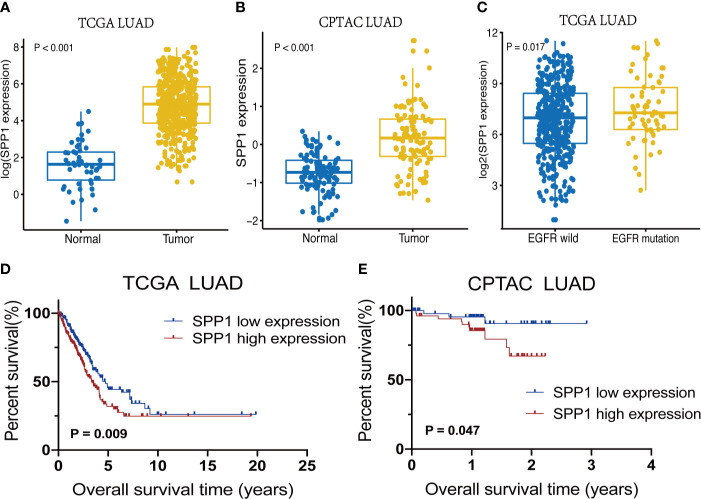
SPP1 expression differences and survival outcomes in LUAD. **(A)** SPP1 RNA expression levels in Normal *vs*. Tumor samples. **(B)** Expression of SPP1 protein in Normal *vs*. Tumor samples. **(C)** SPP1 RNA expression in EGFR wild-type *vs*. EGFR mutant samples. **(D)** Kaplan–Meier survival curves for high and low SPP1 expression groups in TCGA. **(E)** Kaplan–Meier survival curves for high and low SPP1 expression groups in CPTAC.

LUAD cohorts consisted of a total of 477 patients in TCGA and 109 patients in CPTAC. The clinical characteristics between low and high SPP1 expression groups were listed in **Table 1** and **Supplementary Table 1**. SPP1 expression was significantly higher in tumor tissues than in adjacent tissues, regardless of RNA or protein level (**Figures 2A, B**, *P* < 0.001). SPP1 expression was higher in EGFR-mutated tumor samples than in wild-type samples (**Figure 2C**, *P* = 0.017). The median level of SPP1 expression was used to dichotomize patients into high- or low- expression groups, and Kaplan-Meier survival analysis was performed separately for TCGA and CPTAC. Increased SPP1 expression was significantly correlated with poor OS, and the median OS of the TCGA cohort was 4.73 *vs*. 3.37 (**Figure 2D**; HR:1.48 95%CI 1.10-2.00; *P*= 0.009). Median OS was not achieved in CPTAC cohort, but differences were observed (**Figure 2E**; HR: 3.40 95%CI 1.15-10.08; *P* = 0.047). In addition, subgroup analysis of TCGA LUAD showed that patients with EGFR mutation in SPP1 high-expression group had a poor prognosis (**Supplementary Figure 1A**; HR: 1.62 95%CI 0.90-2.93; *P* = 0.055). There was no difference in survival between wild and mutant EGFR patients in SPP1 low-expression group (**Supplementary Figure 1B**; HR: 1.17 95%CI 0.56-2.44; *P* = 0.662).

**Table 1:** The clinical characteristics of patients in TCGA.

	Total	SPP1 low expression	SPP1 high expression	P-value
**Patients**	477	238	239	–
**Age**				0.853
≤65	224 (49%)	111 (48%)	113 (50%)	
>65	234 (51%)	119 (52%)	115 (50%)	
**Gender**				0.967
female	260 (55%)	129 (54%)	131 (55%)	
male	217 (45%)	109 (46%)	108 (45%)	
**Stage**				0.291
stageI	257 (55%)	138 (59%)	119 (51%)	
stageII	109 (23%)	51 (22%)	58 (25%)	
stageIII	78 (17%)	33 (14%)	45 (19%)	
stageIV	25 (5%)	12 (5%)	13 (6%)	
**T stage**				0.38
T1	159 (34%)	86 (36%)	73 (31%)	
T2	255 (54%)	120 (51%)	135 (57%)	
T3+T4	60 (13%)	30 (13%)	30 (13%)	
**N stage**				0.005
N0	307 (66%)	169 (73%)	138 (59%)	
N1	87 (19%)	34 (15%)	53 (23%)	
N2+N3	71 (15%)	28 (12%)	43 (18%)	
**M stage**				0.349
M0	324 (68%)	155 (66%)	169 (71%)	
M1	24 (5%)	11 (5%)	13 (5%)	
Mx	125 (26%)	69 (29%)	56 (24%)	
**EGFR**				0.176
wild	409 (87%)	210 (89%)	199 (84%)	
mutation	63 (13%)	26 (11%)	37 (16%)	

### Identification of Independent Prognostic Factors

**Figure 3:**
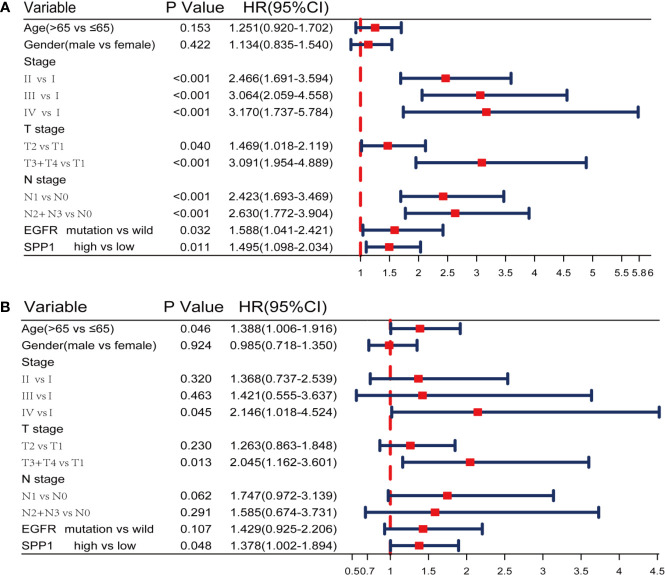
SPP1 was an independent prognostic biomarker in TCGA. **(A, B)** Univariate and Multivariate Cox analysis of SPP1 expression and other clinicopathological factors.

A Cox proportional hazards model including differentiation age, gender, stage, T stage, N stage, EGFR status and SPP1 expression was used. The resulted of univariate and multivariate analysis revealed that different SPP1 expression in patients was significantly associated with OS. Moreover, early tumor stage, and T stage are also the independent factors of favorable prognosis (**Figures 3A, B**).

### SPP1 Expression Mediates Immune Escape

**Figure 4:**
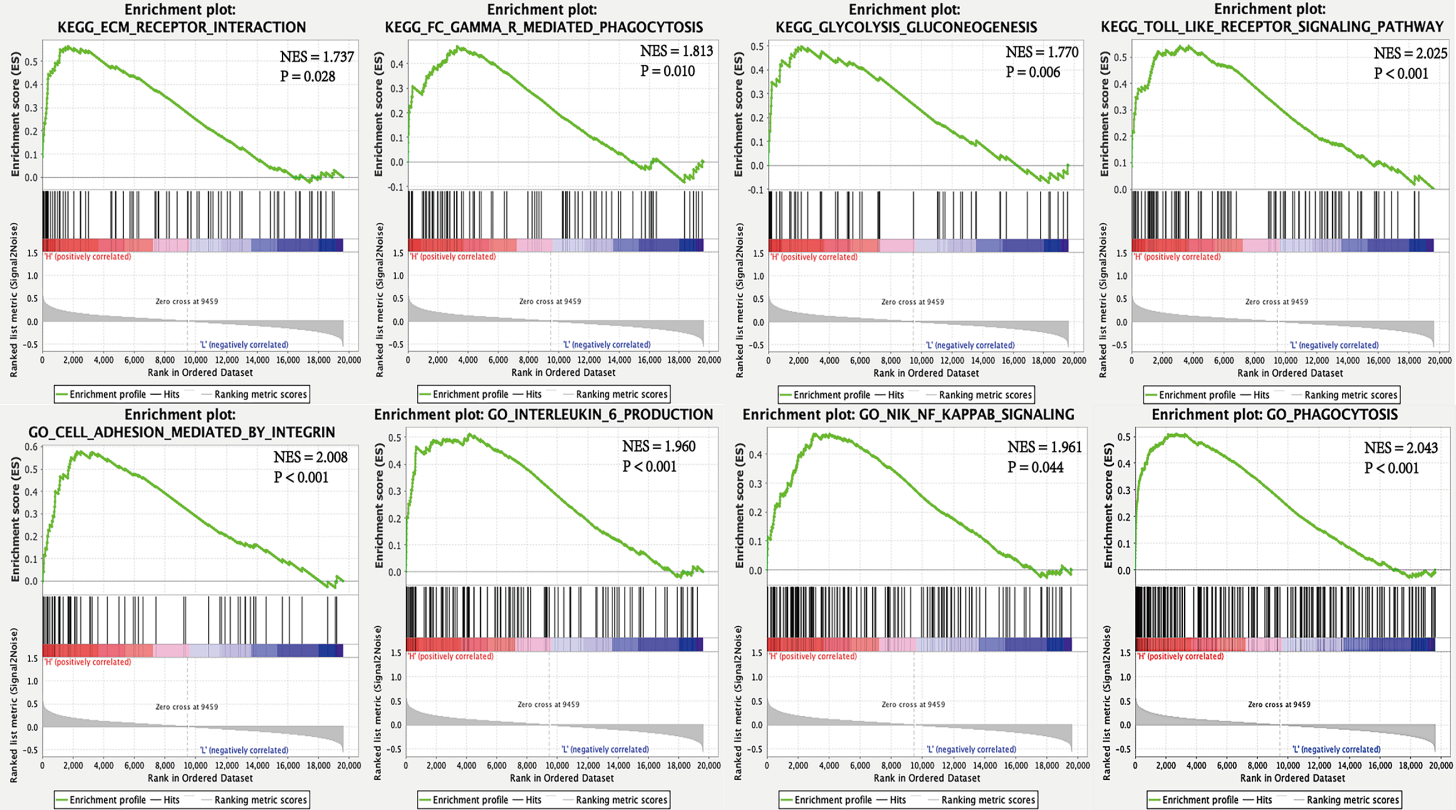
GSEA for high and low SPP1 expression samples.

To interrogate potential signaling pathways related to SPP1 gene in LUAD, we used GSEA analysis (**Figure 4**). We found that the high SPP1 expression group was significantly associated with extracellular matrix (ECM) receptor interaction (NES = 1.737, *P* = 0.028), Fc gamma r mediated phagocytosis (NES = 1.813, *P* = 0.010), glycolysis and gluconeogenesis (NES = 1.770, *P* = 0.006), and the Toll like receptor (TLR) signaling pathway (NES = 2.025, *P* < 0.001). Meanwhile, GO analysis showed that the high SPP1 expression group was positively associated with integrin-mediated cell adhesion (NES = 2.008, *P* < 0.001), interleukin 6 production (NES = 1.960, *P* < 0.001), Nik Nf-Kappa B (NF-κB) signaling (NES = 1.961, *P* = 0.044), and phagocytosis (NES = 2.043, *P* < 0.001).

### Effect of SPP1 on TME

**Figure 5:**
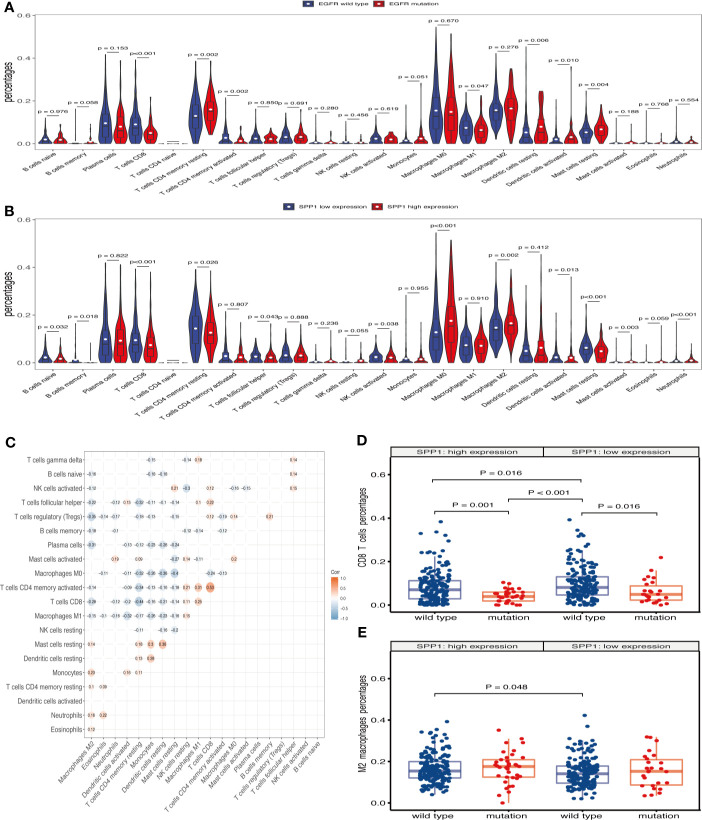
SPP1-related immune infiltration alteration. **(A)** Violin plot showing the ratio differentiation of 22 kinds of TICs in EGFR wild-type and mutant samples. Wilcoxon rank sum was used for the significance test. **(B)** Violin plot showing the ratio differentiation of 22 kinds of TICs in low and high SPP1 expression groups. **(C)** The correlation between different TICs subpopulations. **(D)** Differences in CD8+ T cells infiltration between EGFR mutation and wild-type patients with SPP1 high- or low-expression group. **(E)** Differences in M2 macrophages infiltration between EGFR mutation and wild-type patients with SPP1 high- or low-expression group.

Previous analyses suggested that TICs could be markers of response to ICIs in several cancers (28). In this study, we examined how EGFR mutation and SPP1 expression are related to immune infiltration in LUAD. TCGA LUAD tumor samples (n = 477) were analyzed by CIBERSORT and 368 cases in the wild-type group and 63 cases in the mutant group met the CIBERSORT screening criteria. The results showed that EGFR mutations contribute to reducing the infiltration of CD4+ T cells, CD8+ T cells, M1 macrophages and other immune effector cells in the TME (**Figure 5A**). Taken together, these results indicate that EGFR mutations confer immunosuppressive effects. Additionally, 207 cases in the SPP1 low-expression group and 233 cases in the SPP1 high-expression group met the screening criteria. The results show that high SPP1 expression may play a role in regulating macrophage polarization to the M2 phenotype, reducing TICs such as CD8+ T cells, B cells, follicular helper T cells, NK cells, and activated dendritic cells (**Figure 5B**). These results support the contentions that SPP1 promotes host tumor immune tolerance and immune escape. The correlation heat map (**Figure 5C**) reveals that the different TIC subpopulations are weakly or moderately correlated. Subgroup analysis showed that the proportion of CD8+ T cells infiltration was the highest in patients with wild-type EGFR in SPP1 low-expression group, and the lowest in patients with EGFR mutation in SPP1 high-expression group (**Figure 5D**, P < 0.001). The infiltration of M2 macrophages was the most in patients with EGFR mutation in SPP1 high-expression group, and the least in patients with wild type EGFR in SPP1 low-expression group (**Figure 5E**, P = 0.071).

**Figure 6:**
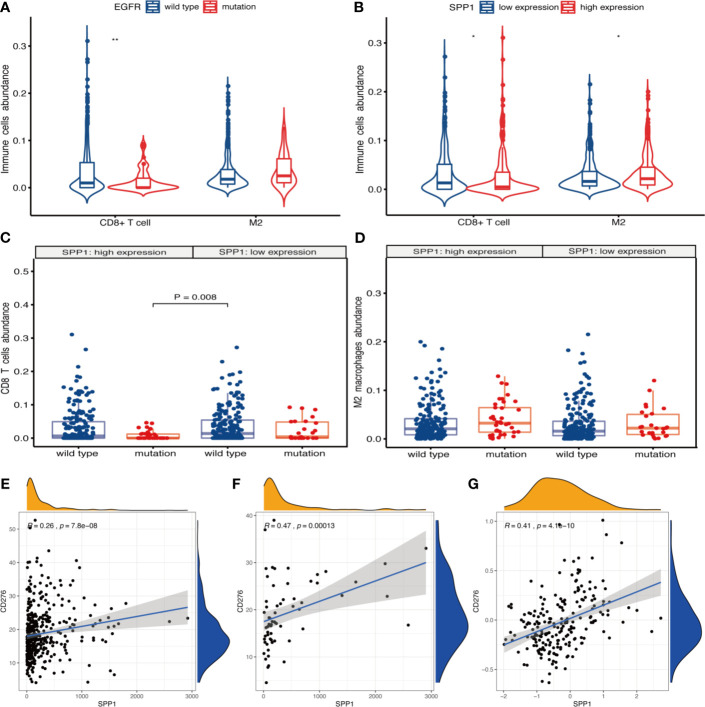
SPP1 promotes immunosuppressive microenvironment in patients with EGFR mutation. **(A)** xCell calculated the abundance of CD8+ T cells and M2 macrophages in EGFR wild-type and mutant samples. **(B)** The abundance of CD8+ T cells and M2 macrophages in different SPP1 expression groups were evaluated by xCell. **(C)** Differences in CD8+ T cells abundance between EGFR mutation and wild-type patients with SPP1 high- or low-expression group. **(D)** Differences in M2 macrophages abundance between EGFR mutation and wild-type patients with SPP1 high- or low-expression group. **(E)** The expression of SPP1 was correlated with CD276 in EGFR wild-type patients at mRNA level. **(F)** The expression of SPP1 was correlated with CD276 in EGFR mutation patients at mRNA level. **(G)** The expression of SPP1 was correlated with CD276 at the protein level. *P < 0.05, **P < 0.01.

xCell results showed that the abundance of CD8+ T cells was less (P = 0.009) and M2 macrophages was more (P = 0.073) in patients with EGFR mutation (**Figure 6A**). There were more CD8+ T cells (P = 0.036) and less M2 macrophages (P = 0.018) in SPP1 low-expression group (**Figure 6B**). The abundance of CD8+ T cells was the highest in EGFR wild-type patients with SPP1 low-expression group (**Figure 6C**). M2-type macrophages in SPP1 high-expression group with EGFR mutation was higher than those of the group with SPP1 low-expression and EGFR wild type (**Figure 6D**, P = 0.073). And we compared CD8 and M2 between the group (SPP1 high and EGFR mutation) and the group (SPP1 low and EGFR wild) in supplement **Figure 2**. Moreover, we found that the SPP1 expression was positively correlated with CD276, especially in patients with EGFR mutation (**Figures 6E–G**).

## Discussion

Immunotherapy can eliminate tumor cells through the body’s immune system and bring long-term survival benefits to patients with NSCLC, significantly ushering in a new era of antitumor therapy. EGFR mutation is a predictor of the therapeutic effects of EGFR-TKIs in patients with LUAD (2, 3). However, it was once considered as a marker of immune resistance (9, 29–31). Here, we recognized the characteristics of TICs in LUAD using bioinformatics analysis. The immune-tolerant TME is more likely to correlate with patients harboring EGFR mutation. Additionally, the observed outcomes indicate that SPP1 may be a potential indicator for patients nonresponsive to ICIs.

In this study, we showed that the SPP1 expression is significantly higher in LUAD tumor tissues and in patients with EGFR mutation. Several previous studies showed SPP1 expression is directly related to CD8+ T cell activation (32, 33), and M2 macrophage polarization (15). Consistently, we found that SPP1 expression was negatively correlated with CD8+ T cell numbers and positively correlated with M2 macrophage numbers. By applying two methods for immunocyte enrichment or proportion analysis, we can find that M2 macrophages enriched more (CD8+ T cells enriched less) in SPP1 high group or EGFR mutation group, and the phenomenon seems more obvious in the group with both SPP1 high and EGFR mutation, which indicated that tumors with both SPP1 high and EGFR mutation tend to show immune evasion phenotype. Therefore, we speculate that SPP1 might cause immune resistance in NSCLC with EGFR mutation.

OPN involved in many physiological and pathological processes, including inflammatory, angiogenesis, tumor metastasis, immune suppression in TME (34, 35). GSEA analysis results indicate the involvement of extracellular matrix (ECM) receptor interaction, Fc gamma r mediated phagocytosis, TLR signaling pathway, integrin- mediated cell adhesion, interleukin 6 production, NF-κB signaling, and phagocytosis in the high SPP1 expression group. OPN is an important component of ECM, regulating matrix interactions and cell adhesion (13). It plays a key role in tumor cell migration by interacting with integrins and CD44 (36). The interaction between OPN and CD44 transmembrane glycoprotein suppressed the CD8+ T cell activation and IFN-γ production. OPN regulation by TLR and NF-κB signaling can reshape the immune inflammatory environment (37, 38). IL-6 binds to IL-6R and activates the Janus kinase (JAK)-STAT3 pathway and the JAK-SHP-2-mitogen-activated protein (MAP) kinase pathway *via* gp130 (39). Previously, it was shown that activation of the JAK/STAT3 pathway can suppress the immune response (40) and that this pathway is activated in patients with EGFR mutation (41). However, it is not clear whether anti-OPN agents, combined with ICIs, can reverse primary resistance in EGFR mutated NSCLC. Therefore, increased SPP1 expression is consistent with a role in immunosuppression, indicating a possible mechanism through which ICIs is ineffective in EGFR-mutated NSCLC.

Pre-clinical studies, involving co-culture of tumor cells and peripheral blood mononuclear cells, show that combined EGFR-TKI and anti-PD-1 antibody therapies do not produce synergistic tumor cell killing effects (42). Multiple clinical studies of EGFR-TKIs combined with ICIs were terminated due to poor efficacy or severe toxicity (43). As a target antibody of vascular endothelial growth factor (VEGF), bevacizumab not only has the effect of anti-angiogenesis, but promotes T cell activation and invasion in tumor tissues (44). Combined with other immunoregulatory drugs, ICIs could be more efficient for the treatment of EGFR-TKI resistance in patients with EGFR mutation. OPN act as an important chemokine and contributes to immune suppression in human colon cancer and other cancers (14, 30). CIBERSORT analysis indicated a significant relationship between SPP1 expression and increased levels of M2 macrophages infiltration, and reduced CD8 + T cell, activated NK cell and activated dendritic cell infiltration. Furthermore, the similarities between CD276 and other immune checkpoints (PD-1/PD-L1, CTLA4) have led to the targeting of CD276 in novel immunotherapy strategy (16, 17). We propose that a combination regimen using anti-OPN and ICIs may be a promising treatment option for LUAD with EGFR mutation, especially with high expression of SPP1. However, further studies should be conducted.

In summary, differences in TICs in patients with EGFR mutation and those with wild-type LUAD may affect the efficacy of ICIs. We observed that LUAD with EGFR mutated have less infiltration of anti-tumor immune cells, including CD8+ T cells, activated CD4+ T cells and M1 macrophages, and increased M2 macrophages infiltration. However, SPP1 likely has an essential influence on TICs, though combined with anti-OPN therapy, has the potential to reverse immune resistance in LUAD with EGFR mutation.

## Data Availability Statement

Publicly available datasets were analyzed in this study. This data can be found here: https://portal.gdc.cancer.gov/, https://proteomics.cancer.gov/programs/cptac.

## Author Contributions

YaZ and SH found the clinical problem and proposed the research direction. YiZ made use of the public databases for bioinformatics analysis. CX and YH searched literatures and analyzed data results. YS and QZ contributed to the original writing of the manuscript and data consolidation. YYZ and MZ undertook the task of modifying the manuscript. All authors contributed to the article and approved the submitted version.

## Funding

This work was supported by grants from the Natural Science Foundation of Hebei Province, China (H2019106033).

## Conflict of Interest

The authors declare that the research was conducted in the absence of any commercial or financial relationships that could be construed as a potential conflict of interest.

## Supplementary Material

Supplementary Figure 1 Effects of EGFR mutation and wild-type on prognosis of LUAD in different SPP1 expression groups in TCGA database. **(A)** Prognosis comparison of EGFR wild-type and mutant patients in SPP1 high expression group. **(B)** Prognosis comparison of EGFR wild-type and mutant patients in SPP1 low expression group.

Supplementary Figure 2 CD8+ T cells and M2 macrophage differed between groups with high SPP1 expression and EGFR mutation and those with low SPP1 expression and wild-type EGFR. **(A)** Differences in CD8+ T cells infiltration calculated by CIBERSORT between SPP1 high-expression with EGFR mutation and SPP1 low-expression with EGFR wild type. **(B)** Differences in M2 macrophages infiltration calculated by CIBERSORT between SPP1 high-expression with EGFR mutation and SPP1 low-expression with EGFR wild type. **(C)** The difference in CD8+ T cells abundance assessed by xCell was compared between the group of SPP1 high-expression with EGFR mutation and SPP1 low-expression with EGFR wild type samples. **(D)** The difference in M2 macrophage abundance assessed by xCell was compared between the group of SPP1 high-expression with EGFR mutation and SPP1 low-expression with EGFR wild type samples.
