# Supplementary figures and images for: The Correlation Between SPP1 and Immune Escape of EGFR Mutant Lung Adenocarcinoma Was Explored by Bioinformatics Analysis
Source: Front Oncol. 2021 Jun 10;11:592854. doi: 10.3389/fonc.2021.592854 (PMC8222997; doi:10.3389/fonc.2021.592854)

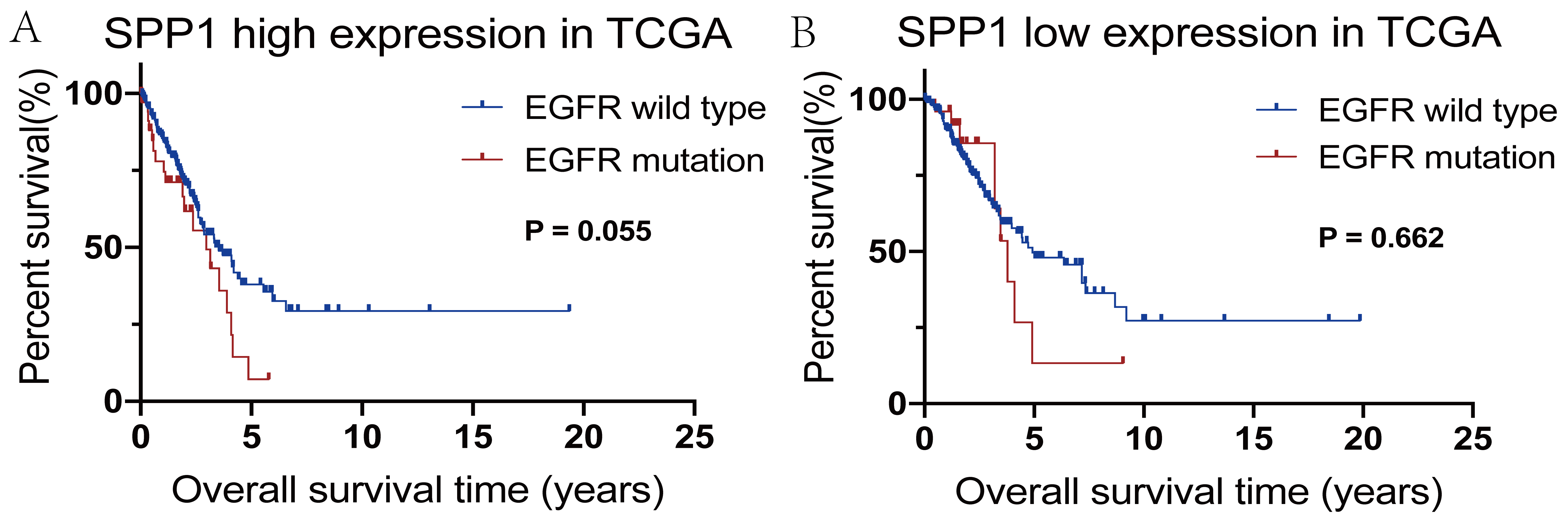

Supplement: Supplementary Figure 1 — Effects of EGFR mutation and wild-type on prognosis of LUAD in different SPP1 expression groups in TCGA database. (A) Prognosis comparison of EGFR wild-type and mutant patients in SPP1 high expression group. (B) Prognosis comparison of EGFR wild-type and mutant patients in SPP1 low expression group. [file Image_1.tif]

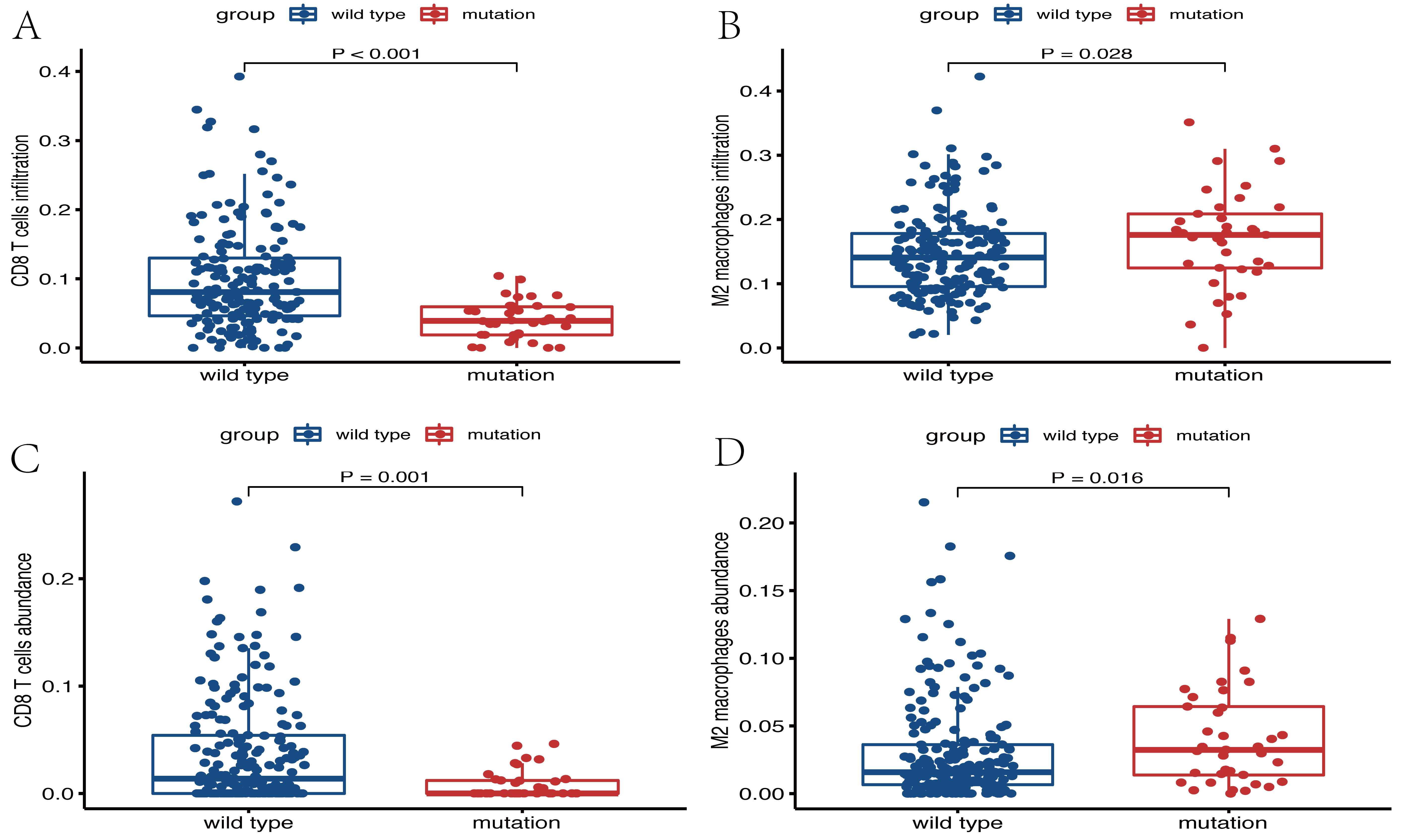

Supplement: Supplementary Figure 2 — CD8+ T cells and M2 macrophage differed between groups with high SPP1 expression and EGFR mutation and those with low SPP1 expression and wild-type EGFR. (A) Differences in CD8+ T cells infiltration calculated by CIBERSORT between SPP1 high-expression with EGFR mutation and SPP1 low-expression with EGFR wild type. (B) Differences in M2 macrophages infiltration calculated by CIBERSORT between SPP1 high-expression with EGFR mutation and SPP1 low-expression with EGFR wild type. (C) The difference in CD8+ T cells abundance assessed by xCell was compared between the group of SPP1 high-expression with EGFR mutation and SPP1 low-expression with EGFR wild type samples. (D) The difference in M2 macrophage abundance assessed by xCell was compared between the group of SPP1 high-expression with EGFR mutation and SPP1 low-expression with EGFR wild type samples. [file Image_2.tif]
